# Expert opinion survey on Israel’s food system: implications for food and health policies

**DOI:** 10.1186/s13584-024-00590-3

**Published:** 2024-01-15

**Authors:** Emily Soh, Elliot M. Berry, Eran Feitelson

**Affiliations:** 1 Department of Geography, Hebrew University of Jerusalem, Jerusalem, Israel; 2 Braun School of Public Health, Hebrew University Hadassah Medical School: Hebrew University of Jerusalem School of Medicine, Jerusalem, Israel

**Keywords:** Expert opinion survey, Food system transitions, Food policies, Food security, Sustainability, Nutritional security

## Abstract

**Background:**

While there has been increasing global recognition and impetus for action to transform food systems towards greater food security, sustainability and better health outcomes, Israel has only recently begun to focus on the diverse challenges of its food system and its potential for transformation.

**Methods:**

An expert opinion survey (n = 50) on Israel’s food system was conducted as part of a larger study on the systemic features of Israel’s food system transition to understand its policy gaps and find strategies towards a healthy and sustainable food system. The survey ranks the relevance and importance of food system challenges and policy preferences. Policy implications are then examined by identifying potential priorities, gaps and dissensus.

**Results:**

The survey finds that there is a majority agreement (76%) that Israel’s food policies are lacking or severely lacking. Respondents relate strongly to both concepts of nutritional security (90% think that access to nutritious food is relevant or highly relevant) and national food security (more than 80% perceive food security as part of national security). Respondents overwhelmingly recognize the benefits of Israeli agriculture with 60–90% agreeing or strongly agreeing that it benefits food security, economic value and national identity. Top-ranked problems include overall systemic problems such as the lack of national goals, strategic planning, and integrated policymaking across ministries, and specific ones such as food waste, costly farming inputs, and food affordability. The most preferred policy actions include establishing a national strategy for food and agriculture, making food affordable for vulnerable households, and incentivising sustainable farming methods. The key policy gaps include the lack of resilience in agriculture and the food system, insufficient data and knowledge for policy action, inadequate attention to the regulation of the food industry for better health and inadequate food policy attention for minority groups.

**Conclusions:**

Building on this study's findings, further policy research and implementation areas to be covered include government responsibility for universal food security, strategic systemic policies for food systems, prevention and preparedness for future crises, and promoting resilience. The way forward may best be through an inter-ministerial committee with the responsibility, budgets, mandate and executive authority to plan data-driven policies for a sustainable food system for Israel’s future.

## Introduction/background

There is increasing global recognition and impetus for action to transform food systems towards greater food security, sustainability and better health outcomes [34], while aspiring to achieve developmental objectives in equity, inclusivity, resilience, and efficiency [36]. Israel’s food system, however, is yet to be adequately prepared to deal with urgent (rising food prices, uneven access to affordable and healthy food, and nutritional insecurity), and longer-term issues (strategic plan for agricultural production, food system sustainability, climate change impacts on food security, geo-political upheavals, etc.) [1]. The outbreak of the war in October 2023 is expected to aggravate food insecurity with a current loss of 40% of the agricultural workforce, restricted access to 30% of agricultural lands as they are located in the Gaza envelope, and a price hike of agricultural produce [20].

Recent works addressing diverse areas in Israel’s food system include food security [13], food welfare, food prices and economic policy [4], agricultural production [17], food waste [25], food and public health [9, 14], environmental impacts and sustainability [12, 30, 33], and food systems and society [15]. However, there are insufficient academic studies addressing Israel’s food system in an integrated and multidisciplinary manner. Some examples of such studies in the international arena include transitioning food systems toward a circular economy [16], scaling pathways in food system transitions [26], and positioning local resilience and global engagements in food system transformations [27].

This paper on an expert opinion survey on Israel’s food system contributes to addressing these gaps. The survey was conducted in 2022 as part of a larger study on the systemic features of Israel’s food system transition to understand policy gaps and find pathways towards a healthy and sustainable food system. This survey is structured on the findings of initial interviews (not covered here) with food and agricultural practitioners and researchers to determine the relevant subjects to be covered by the study. The survey ranks the relevance and importance of food system challenges and policy preferences of respondents. We then discuss the policy implications by examining potential policy priorities, gaps and dissensus raised from the results. Through this, we seek to contribute to policy discussions towards a more integrated, healthy, and sustainable future for Israel’s food system.

## Methodology

While this paper focuses on the findings of an online survey (n = 50), it benefitted from a set of in-depth interviews (n = 17) carried out shortly before the survey. Both the interviews and survey sought “expert” respondents who are Israeli food and agriculture practitioners and researchers in the broad domains of food security, agriculture, health and nutrition, governance and policymaking, food and society, environmental sustainability, and technology. Some interview respondents also did the survey (but the numbers are unknown as survey respondents may choose to remain anonymous). The study did not relate to seniority in profession or age, educational qualifications, or number of years in the profession. We searched for respondents on the internet and from contacts known or recommended to us and emailed them the survey links. The email invitation also requests that the survey be forwarded to others in the field of food and agriculture, thereby initiating a respondent-driven snowball sample [24].

The interview findings and relevant literature were used to build the survey questions. The survey comprises mainly quantitative Likert scale questions, each followed by open-ended answer boxes for optional additional comments. Participants could choose to do the online survey in English or Hebrew.

To analyse the findings, we used a 5-point Likert scale for both unipolar and bipolar questions. Unipolar questions give a range of options based on the degree of a single characteristic, for instance ranging from “not important at all” to “very important”. Bipolar questions have two opposite ends such as “strongly disagree” and “strongly agree” with a neutral option in between. The responses were weighted for the bipolar questions (strongly agree—5 points; agree—4; neutral—3; disagree—2; strongly disagree—1), and for the unipolar questions (very important—5 points; important—4; moderately important—3; slightly important—2; not important at all—1). The total scores were then ranked in descending order and presented in charts (Figs. 3, 4, 5, 6, 7, 8, 9, 10). The open-ended responses were examined through thematic analyses to identify additional relevant topics such as education, sustainable agriculture, and resilience in the food system (elaborated below) raised by the respondents.

**Fig. 1:**
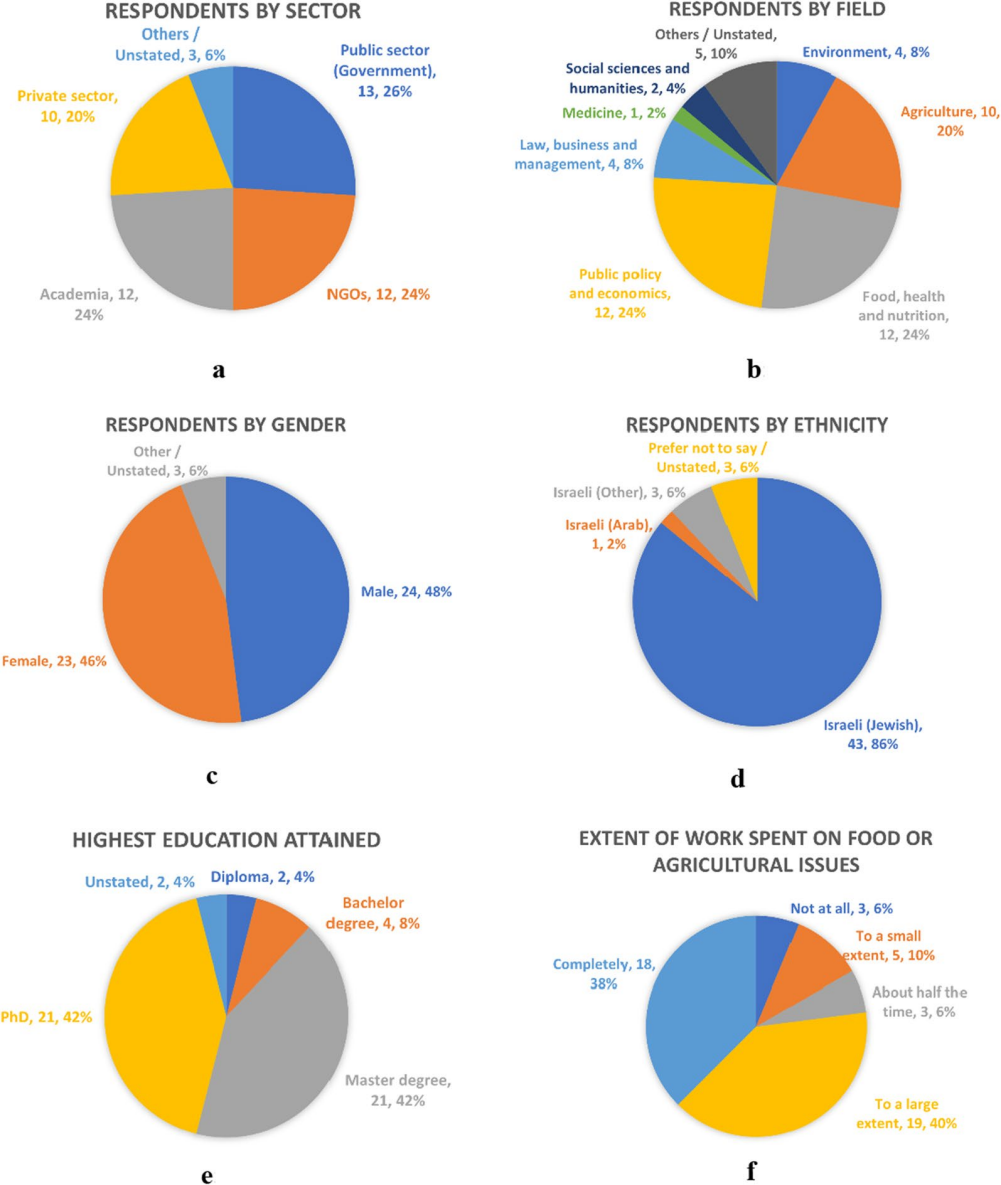
**a** Respondents by Sector. **b** Respondents by Field. **c** Respondents by Gender. **d** Respondents by Ethnicity. **e** Respondents by Highest Education Attained. **f** Respondents by Extent of Work Spent on Food or Agriculture Issues

The characteristics of the respondents are shown in Fig. 1a–f. The respondents were quite evenly spread out by the four sectors—public and private sectors, NGOs, and academia (Fig. 1a). Respondents came from a variety of fields, the three largest were “food, health and nutrition” (24%), “public policy and economics” and “agriculture” (20%) (Fig. 1b). They were quite equally divided by gender (Fig. 1c), but Jewish respondents were over-represented (86%, compared to their 76% share of the national population). Arabs were very under-represented, (2%, compared to their 21% share of the national population) (Fig. 1d). There could be several reasons for this. One was that the survey was not translated into Arabic. It can be noted that out of the 50 survey responses, only 5 respondents chose to answer in English while 45 chose the Hebrew survey. Another reason could be that there are fewer Arab experts in food and agriculture. In our search for Arab interview respondents, we also encountered difficulties finding any, although there were Jewish respondents who worked extensively with the Arab population. 84% of the respondents have postgraduate degrees (Fig. 1e), which may indicate that many are professionals. This aligns with the survey’s intention to seek informed responses through practitioners and researchers in food and agriculture. 78% of the respondents worked completely or to a large extent on food and agriculture issues (Fig. 1f), which indicates a high level of familiarity with the topic.

## Results

### Overall opinion on food policies, prices and food security

**Fig. 2:**
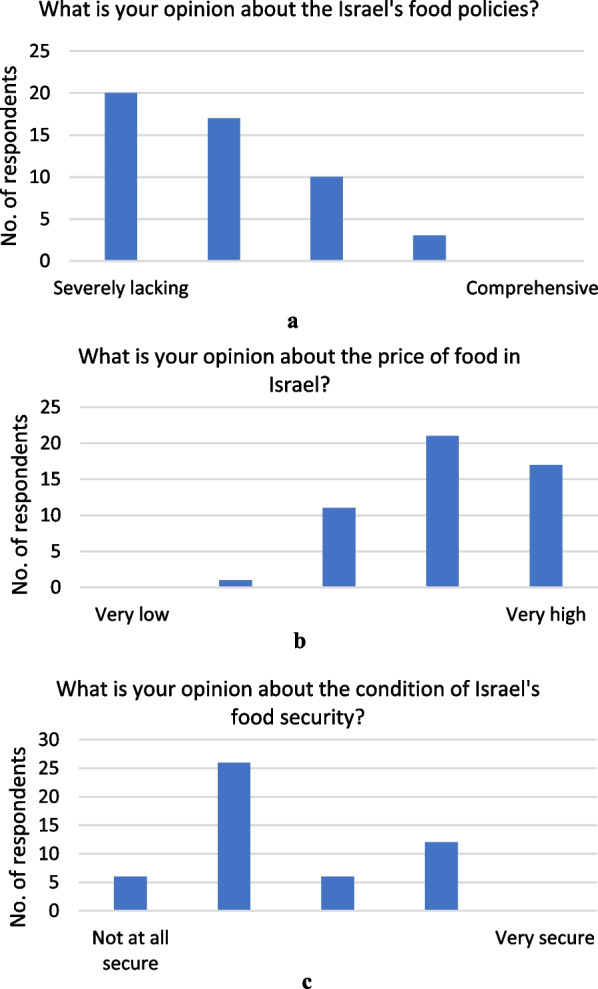
**a** Opinion on sufficiency of Israel’s food policies. **b** Opinion on price of food. **c** Opinion on condition of food security

Figure 2a–c show respondents’ opinions on three facets of the system: the sufficiency of Israel’s food policies, the price of food and the condition of food security. There is a majority agreement (76%) that Israel’s food policies are lacking or severely lacking. Similarly, 76% think that the price of food is high or very high in Israel. The responses for the state of food security are more divided with 64% thinking that Israel is not food secure or not food secure at all, and 36% with the opinion that it is secure or very secure.

### Opinion on food security

**Fig. 3:**
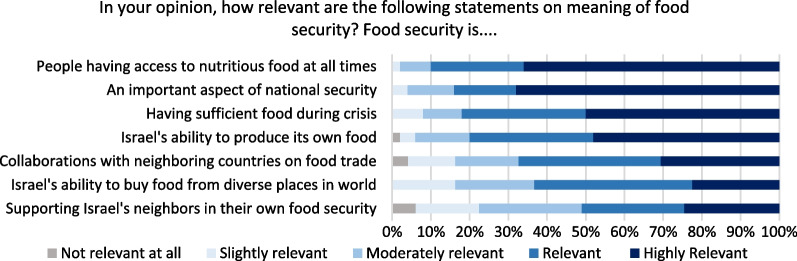
Opinion on meaning of food security

Respondents related strongly to both concepts of nutritional security (at individual and household levels) and national food security. Figure 3 shows 90% of respondents deem access to nutritious food as relevant or highly relevant. More than 80% perceive food security as part of national security and having sufficient food during times of crisis as relevant or highly relevant. In comparison, just over 60% think that Israel’s ability to import food is relevant or highly relevant. This is surprising because Israel imports 55% of its caloric food supply [18], with high import dependency for food items such as cereals and cereal products (97% imported), fish (91%), and legumes, peanuts and nuts (75%) ([7]: 19). In the open-ended responses, several argued for the importance of local food production for food security. One such response was, “Food security is the state's ability to provide a supply of quality and healthy food at fair prices for every person over time. Looking ahead, maintaining the autonomy of food production cannot be avoided as a condition for ensuring food security”.

### Opinion on the state of agriculture in Israel

**Fig. 4:**
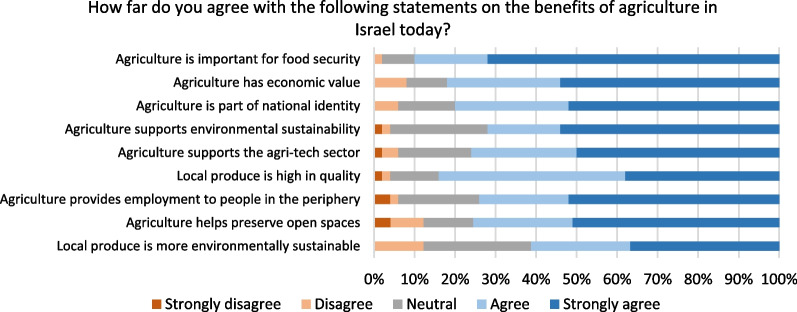
Opinion on benefits of agriculture in Israel today

Respondents overwhelmingly recognize the benefits of Israeli agriculture, with 60–90% agreeing or strongly agreeing that it benefits food security, economic value, national identity and more (Fig. 4). A few open-ended responses noted that local agriculture contributes to stability in the food system, as one commented, “Food policy must account not only for the current price of food but also the risks of food systems in the world, including increasing competition for food, risks from climate change, and damage to agricultural land and ecosystems”. Another respondent emphasized the reality that “Israeli agriculture cannot guarantee food security under any conditions, except for vegetables and fruits”.

**Fig. 5:**
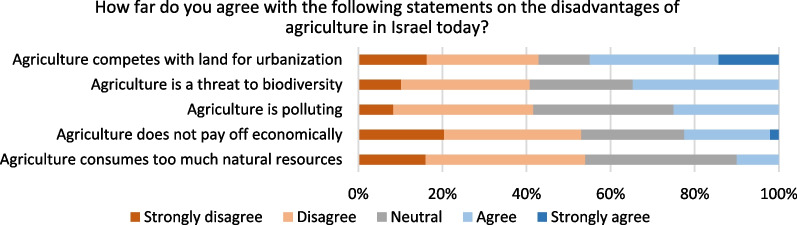
Opinion on disadvantages of agriculture in Israel today

Opinions on the disadvantages of agriculture are more divided, with about 40–55% of responses disagreeing or strongly disagreeing with statements such as agriculture consumes too many resources, does not pay off economically or is polluting (Fig. 5). In the open-ended answers, respondents suggest that the negative effects of agriculture can be mitigated, and that the kind of practices implemented determine whether agriculture is environmentally detrimental and not the existence of agriculture itself. Others suggest there is scope for improving the environmental performance of agriculture in Israel, including a transition to sustainable agriculture, examining effects and policies on soil pollution and chemical pesticides, and animal agriculture. On the economic value of agriculture, one noted that “agriculture as a political resource [that] cannot be measured based on national profit/loss”.

**Fig. 6:**
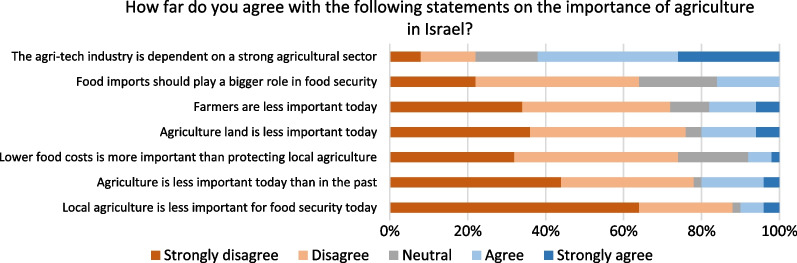
Opinion on importance of agriculture in Israel

Figure 6 shows ranked opinions on the different facets of the importance of agriculture in Israel. The questions juxtapose various priorities vis-à-vis domestic agriculture, such as food affordability and the role of food import for food security. 74% disagree or strongly disagree that lower food costs are more important than protecting local agriculture, and 64% disagree or strongly disagree that food imports should play a bigger role in food security. This could signal a lack of agreement with the agricultural reforms’ way of implementation or its goals to increase competition and lower the cost of food by increasing imports. The reforms were announced in July 2021, with the first tranche of import tax abolished on selected fruits and vegetables implemented in 2022 [22]. Notably, 88% disagree or strongly disagree that local agriculture is less important for food security today than in the past. That is, it seems the experts strongly disagree with the recent market-oriented policies, mainly advanced by the Treasury to lower consumer prices. In the open-ended responses, several disagreed with framing food affordability and local agriculture as opposing goals, as one stated, “It is wrong to pit local agriculture against the price of food. Promoting and strengthening agriculture should lead to more stable and less expensive food systems. The state should invest in agriculture to strengthen its benefits, but not at the expense of higher prices”.

### Opinion on Israel’s food system

**Fig. 7:**
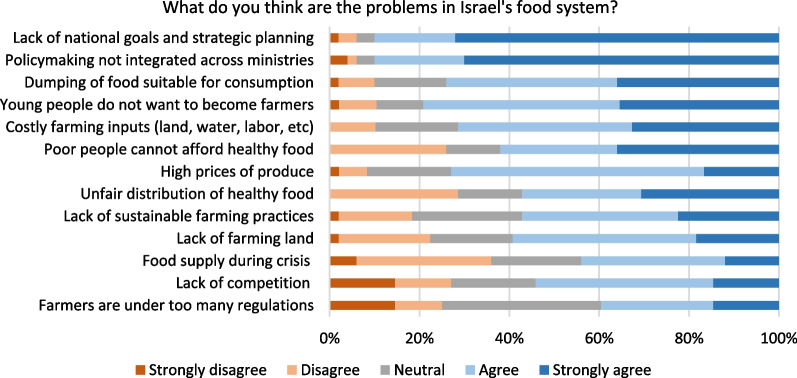
Opinion on problems of Israel’s food system today

Figure 7 shows the top-ranked problems (over 90% agreeing or strongly agreeing) are, the lack of national goals and strategic planning, and a lack of integrated policymaking across ministries. While these two are overall systemic problems, the rest are specific ones such as food waste, costly farming inputs, and food affordability. Most of these issues are perceived as problems, except two to a lesser extent—food supply during a crisis and farmers subject to too many regulations received less than 50% affirmative response. Other than the problems stated in the survey, a respondent also added, “There is no organized database in agriculture, what is grown in what quantities, where is it grown, what will be harvested and when; there is no information for both the farmers and the state to plan and prepare”. Another commented on how available resources are not efficiently used, “Only 60% of the land suitable for agriculture is cultivated, so there is no shortage of land; water is not lacking—these are administrative issues”.

### Opinion on food policies

**Fig. 8:**
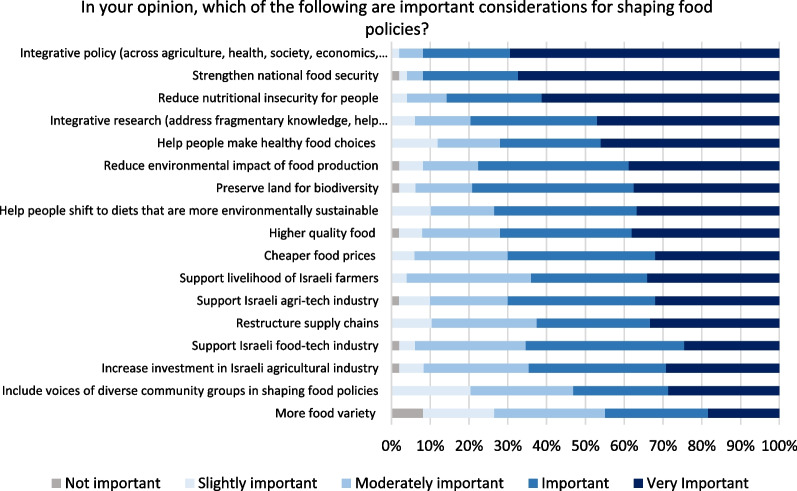
Opinion on considerations shaping food policies

The importance of having integrative policies again features as the most important consideration in food policies (Fig. 8). The highest-ranked specific issues related to strengthening national food security (more than 90% deem it as important or very important), reducing nutritional insecurity (85%), helping people make healthy food choices (72%), and reducing the environmental impact of food production (77%). Socio-cultural issues may be less recognized as the inclusion of diverse voices in food policy is relatively lower ranked (53%).

**Fig. 9:**
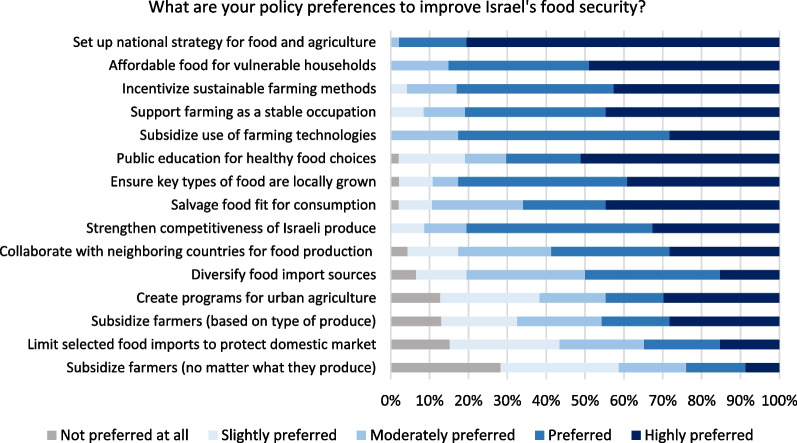
Opinion on policy preferences for food security

Figure 9 on policy preferences reiterates the key relevant policy areas identified in Fig. 8. An overwhelming 97% prefer or highly prefer a national strategy for food and agriculture. Issues of affordability of food, sustainable agriculture, support for agriculture, and nutritional security remain important policy preferences. 50% prefer or highly prefer the policy of diversifying food import sources for food security. This represents a divided opinion over food imports as a food security policy (despite the fact of Israel’s high dependency on food imports). Interestingly, just 35% prefer or highly prefer to limit selected food imports to protect the domestic market. This contradicts the result in Fig. 6 where 64% disagree or strongly disagree that food imports should play a bigger role in food security. A possible interpretation of this contradiction could be that most do not prefer a growing dependence on food imports but they do not prefer to use tools of market protectionism to achieve that end. Otherwise, this could reflect that food import (its role, extent, and policy approaches) is a policy grey area, where its implications are not well-understood.

### Opinion on food (in)justice

**Fig. 10:**
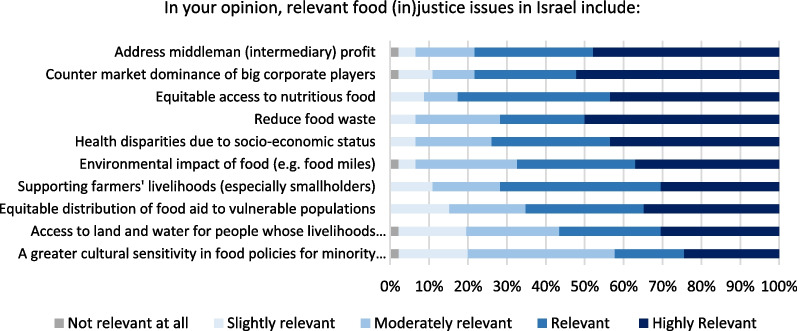
Opinion on food (in)justice in Israel

The top issues of food (in)justice perceived by respondents are intermediary profits (78% think are relevant or highly relevant) and the dominance of large corporate players (78%). This is closely followed by equitable access to nutritious food (Fig. 10). Much lower on the priority list appear to concern minority groups—just over 50% think that access to land and water for people whose livelihoods depend on agriculture/herding is a relevant or highly relevant issue. Notably, just over 40% deem that having a greater cultural sensitivity to minority groups is relevant or highly relevant. This finding resonates with the relatively low ranking of the perceived importance of including diverse voices in food policy (Fig. 8). This may suggest that socio-culturally responsive food policies are lacking, and significant challenges remain in achieving greater inclusivity, as data reflect a consistent pattern of minorities such as Ultra-Orthodox Jews (15.8% in food insecurity in 2021) and Arabs (42.4%) have a much higher rate of food insecurity than the non-Ultra-Orthodox Jews (10.7%) [8]. This could also reflect the composition of the respondents, who were not from minority groups.

### Other themes from the open-ended responses

Some of the respondents raised insights or provided contrary framing of problems in the open-ended responses. These opinions relate to the long-term considerations of the food system. We summarize three such additional themes:

#### Education

Respondents mentioned two aspects of education, first, as a necessary factor to strengthen and renew the domestic agricultural sector. One mentioned that “agriculture should be considered as education not just as another economic sector”. Another felt that “agricultural education should be added to the schools. Something that will allow the students to connect with the subject and perhaps produce the farmers of the future”. The second aspect of education relates to consumer awareness of sustainable consumption. One respondent commented that “we need to do both—both lower prices and develop local agriculture and above all educate the public on economical consumption and waste prevention”. On tackling food waste as part of sustainable consumption, one noted that “food waste is a huge phenomenon that needs to be eradicated. Reducing food waste will contribute to the local economy, stabilizing the food system and its resilience, […] lower the cost of living and national expenditure on food”.

#### Resilience

Respondents relate to various aspects of stability and resilience (the ability to reduce and cope with system vulnerabilities [28], and recover from adverse events). Several respondents questioned the assumption that food affordability and local production are conflicting goals but argued that they can go together through policy planning, as one said, “It is of great importance to create stability in the food chain through local agriculture”. Another remarked, “The notions that strengthening agriculture is at the expense of the price for the consumer, and reducing prices comes at the expense of local agriculture are outdated and oversimplified. The fact is that a strong agricultural sector is important to the country and strengthens the economic system”. These comments touch on the economic stability of food prices for consumers, and the stability of the Israeli agricultural sector.

Some respondents also frame issues in terms of economic and environmental risks, as one noted, “Food policy must consider not only the current price of food but also food systems risks in the world, including increasing competition for food, risks from climate change, and degradation of agricultural land and ecosystems.”. These respondents prioritize the longer-term perspectives by factoring in resilience and risks and see the importance of investing in domestic agriculture for future stability in supply and prices.

#### Sustainable agriculture as the future

Sustainability in the food system refers to the ability to deliver food security and nutrition to all using social, economic and environmental resources in ways that do not compromise the ability of future generations to do so [6, 11]. Several respondents raise the importance of shifting to sustainable agriculture, in part to deal with the negative effects of agriculture (raised in the survey), and in part to improve future practices. A respondent remarked, “Care should be taken to shift agriculture to sustainable practices, but in no way to abolish it”. Another noted that “local agriculture is important for preserving the environment and sustainability values. It is not at all clear that Israeli agriculture in its current state is there”.

## Discussion

This section discusses the policy implications of the findings in three areas: policy priorities, policy gaps and policy grey areas.

### Policy priorities

The survey sought to rank the importance of food system issues as perceived by the respondents, and from these to extrapolate possible policy priorities. We identified three top policy concerns. First is the need for integrative policies cutting across diverse areas of the food system, including food and nutritional security, public health, food welfare and equity, food economics and supply chain, sustainable agriculture, and food waste [23, 36].

Secondly, a strategic and long-term vision is needed for policy prioritization. This helps to differentiate issues that are important or urgent, or both; it also brings a time perspective of the immediate and long-term goals. Evaluating these two dimensions can support clearer policy prioritization, and ensure that short-term goals or policy low-lying fruits (for instance lowering food prices with food imports) do not compromise long-term values and goals (such as the viability of domestic agriculture). More elaborate guidance on policy prioritization can be found, for instance in Taeihagh et al. [32] who advance measurement criteria such as expected cost, effectiveness, the timescale for implementation, technical and institutional complexity, and public acceptability as measures to set priorities.

The dual goals of food affordability and strong domestic agriculture—both perceived as important and may appear contradictory under current conditions—should not just be a balancing act where one prevails at the expense of the other. Some respondents point out that policy should support shifts in both areas so that domestic agriculture supports affordable and healthy food while boosting food and nutritional security. Adding a third goal, sustainable agriculture (highly prioritized by respondents), further increases the complexity with additional trade-offs. A policy roadmap would be needed to integrate important goals and seek their synergies to shift knowledge, practices and technologies [5, 37]. To this end, a systemic national plan– a policy action strongly supported by the respondents—would help to guide policy strategies and interventions in all stages of the food production and consumption chains.

### Policy gaps

Based on the survey, we identify four key policy gaps: the lack of resilience and stability in the agriculture and food system (addressed above); insufficient data and knowledge for policy action; inadequate food policy attention for vulnerable groups; reforming the food industry for better health, equity and sustainability outcomes.

There is insufficient timely and accurate data and knowledge to support decision-making for policymakers and agriculture practitioners. There is a need to (re)establish data infrastructure that includes food system performance and risk indicators (for economic, food security and environmental risks) to measure and enhance its resilience. While comprehensive databases existed in the past when central planning was in vogue in the agricultural sector, the data needed today varies widely from those collected in the past.

There needs to be greater food policy attention for vulnerable groups [8, 29]. An example is the monitoring of micronutrient consumption of population groups such as the elderly, pregnant women, infants and children, and other socio-demographic groups at risk of under- or mal-nutrition. Again, this needs to begin with data and knowledge building. In addition to quantitative data, ethnographic knowledge can bring in cultural perspectives of food production and consumption practices upon which to build culturally sensitive and inclusive policies.

Finally, more policy attention is needed for restructuring and regulating the food industry for better health, equity and sustainability outcomes. This was insufficiently covered in the survey but emerged in the interviews. Issues pertinent to Israel include market consolidation by large players, shifting production towards healthy food and away from ultra-processed food; and addressing decades-long policy inaction on food fortification [9, 31].

### Policy grey areas and dissensus

Our findings show where there are policy grey areas or dissensus. There is an apparent divide over whether Israel is food secure (Fig. 2c). This may be attributed to differences in the way respondents relate to the multifaceted concept of food security. For instance, the Food and Agriculture Organization [10] delineates different dimensions of food security—availability, accessibility, utilization, and stability, while Berry et al. [6] argue for an additional long-term (inter-generational) dimension of “sustainability”. Specific to Israel, a recent Knesset document ‘Food system security in Israel’ mapped the definitions of food security by different government ministries [21]. This document identifies two broad spheres of definitions of food security, roughly corresponding to the spheres of government action. The first is “national food security”, which deals with the sufficiency of food production and supply at the national level. The second is “nutritional security”, which addresses the ability of individuals and households to access and purchase food that meets their nutritional needs for optimal well-being (similar to the USDA definition—USDA website). The two terms in Hebrew are often used interchangeably in the general literature.

The survey did not delve into how respondents define food security, but the different perceptions on whether Israel is food secure may come from the ways respondents relate to the different dimensions of food security, their respective disciplinary lenses, or even relating to different time scales of current or future food security.

Another area of disagreement is over the disadvantages of agriculture in Israel. This may relate to considerations of which agricultural practices should be used to incorporate environmental, political and socioeconomic concerns, as well as how the organization, practices and technologies of agriculture should change in the future. A related policy grey area is the strategy for food import, broaching questions on how to balance food import and domestic production, and whether Israel should main production capacity for a core or essential selection of food crops. Amdor (2022, 2023), for instance, investigated 23 core food items consumed in Israel based on a comparison of their environmental impacts if they were produced in Israel or imported. Other works measuring the environmental impacts of food trade concerning Israel [12, 30] can also be instrumental in making decisions about food imports.

### Study limitations

This survey offers a preliminary assessment of the challenges, potentials and policy implications as perceived by experts in the field. For a more thorough examination of the topic, this research should be complemented by more in-depth qualitative research (for instance, interviews and focus group discussions) and quantitative research (including comprehensive, cross-population and targeted population studies). This study is also limited in its underrepresentation of Arab respondents, as well as providing a more nuanced picture of the opinions of minority groups such as the Ultra-Orthodox and the Bedouin.

## Conclusions

### Implementation recommendations

The survey side-stepped the issue of the ways for the practical implementation of the results in formulating a national policy for sustainable food systems. An example of such a challenge was the distribution of food aid during the recent COVID-19 pandemic. Some eleven government ministries were involved, in addition to many NGOs. This was both costly and inefficient.

Building on this study's findings, further policy research and implementation areas to be covered include government responsibility for universal food security, strategic systemic policies for food systems, prevention and preparedness for future crises, including climate change, and promoting resilience. The way forward should probably best be through an inter-ministerial committee with the responsibility, necessary budgets, mandate and executive authority to plan data-driven policies and priorities to ensure a sustainable food system for the future of Israel. A first step in this direction may be seen in the recent report of the national committee on food systems adaptation and mitigation of climate change [19].

## Data Availability

The datasets during and/or analysed during the current study available from the corresponding author on reasonable request.
